# Edge Computing for Vision-Based, Urban-Insects Traps in the Context of Smart Cities

**DOI:** 10.3390/s22052006

**Published:** 2022-03-04

**Authors:** Ioannis Saradopoulos, Ilyas Potamitis, Stavros Ntalampiras, Antonios I. Konstantaras, Emmanuel N. Antonidakis

**Affiliations:** 1 Department of Electronic Engineering, Hellenic Mediterranean University, 73133 Chania, Greece; ddk86@edu.hmu.gr (I.S.); akonstantaras@hmu.gr (A.I.K.); antonidakis@hmu.gr (E.N.A.); 2 Department of Music Technology and Acoustics, Hellenic Mediterranean University, 74100 Rethymno, Greece; 3 Department of Computer Science, University of Milan, 20133 Milan, Italy; stavros.ntalampiras@unimi.it

**Keywords:** e-traps, pest detection, image sensors, edge computing

## Abstract

Our aim is to promote the widespread use of electronic insect traps that report captured pests to a human-controlled agency. This work reports on edge-computing as applied to camera-based insect traps. We present a low-cost device with high power autonomy and an adequate picture quality that reports an internal image of the trap to a server and counts the insects it contains based on quantized and embedded deep-learning models. The paper compares different aspects of performance of three different edge devices, namely ESP32, Raspberry Pi Model 4 (RPi), and Google Coral, running a deep learning framework (TensorFlow Lite). All edge devices were able to process images and report accuracy in counting exceeding 95%, but at different rates and power consumption. Our findings suggest that ESP32 appears to be the best choice in the context of this application according to our policy for low-cost devices.

## 1. Introduction

Smart cities rely on a range of technologies—including artificial intelligence (AI), the internet of things (IoT), and wireless connectivity solutions to provide social services that promote quality of life and sustainability to their citizens. Sensor technology and AI practices that process these sensors can leverage detection and density estimation of creatures that have attained the pest status in daily practice. This includes rodents spreading through a network of buildings, stinging insects that carry vector-borne diseases (mosquitoes, biting midges), wood-boring insects that can inflict structural damage to wood (termites, wood-boring beetles in urban greenery), sanitary problems in hospitals, schools, metro lines (cockroaches), domestic health threats (bed bugs), or simple annoyance only by the insect presence (ants in houses, clothes moths, spiders, millipedes, centipedes). Pest management and control strategies are based on early detection and pest identification before planning the treatment strategy that includes the application of chemical and nonchemical control treatments. Early pest detection is crucial for effective and affordable control in urban environments, but manual assessment of traps cannot expand in vast spatial and time scales because of cost and manpower constraints.

In recent years, we are witnessing an upsurge of interest in technologically advanced devices as applied to automatic insect detection, counting, and identification [1]. There are mainly three major approaches: (a) optical counters attached to the entrance of traps that target specific pests using lures (pheromones in the case of lepidoptera [2] and palm pests, soil arthropods [3,4] or scents and CO_2_ in the case of mosquitoes [5]), (b) camera-based traps that take a picture of their internal space [6,7,8,9,10,11,12,13,14], and (c) near infrared sensors [15] and lidars that emit light covering a volume of space of the open field and registering the backscattered wingbeat signal of flying insects [16,17,18]. All approaches have advantages and disadvantages depending on the application scenario. In short, optical counters are low-cost, low-power, and count insects upon their entrance into the trap. Therefore, they can count a large number of insects per day as in the case of mosquitoes and lepidoptera (e.g., *Tuta absoluta*, *Helicoverpa armigera*). They do not face luminosity variations or miss overlapping insects (as is the case with camera-traps) or encounter unknown insect fauna (as is the case with lidars). Their disadvantage is that they only sample a targeted subset of insect fauna at specific locations, and one may need a dense network to face the volatility of insect densities. Moreover, the counting modality does not offer the amount of information a picture can provide or the enormous numbers of insects a lidar-based technology can register. If the targeted pest is a single species, then we face a binary problem, and a smart trap may be the best option compared to a costly lidar. If the task involves the continuous and unobtrusive biomonitoring of insect abundance, biomass, and diversity over a field, then lidars have the advantage.

Optical counters rely on the specificity of lures to attribute class to captured insects, whereas camera-based approaches rely on the classification of the contents of the captured image. This work adopts the third approach as it is best suited for crawling/walking Arthropoda encountered in urban environments. There is a large corpus of previous approaches on cameras embedded in insect traps (see [6,7,8,9,10,11,12,13,14] and the references therein). Their role is to either report a picture to a server and let a human observer discern the number and the species of the captured insects [14], or proceed into processing the captured image to detect [10,12] and automatically count and/or identify insects [7,8,9,10,11,13,19]. In the former case, the gain is the reduction of cost and manpower required to visit the network of traps and deliver the photos. However, in the case of dense networks that upload many images, the manual inspection on the server side can become impractical. In the latter case, the image can be processed automatically, relieving the burden of manual identification, but the classification results can be inferior to those detected by a human eye or the manual counting of insects in situ.

An insect in the context of image processing can be seen as a deformable template that can be found oriented at any angle in the trap. We are in line with [1] that this kind of problem is best tackled by deep learning (DL) architectures [19,20,21] that have a modular layer composition where the layers close to the input learn to extract low-level features (e.g., starting from the edges of insect legs/antennas and proceeding to the main body curves) and subsequent layers rely on the previous one(s) to synthesize patterns of higher abstraction and textures (as the texture of wings and body) and ending in insect species [21]. DL [22,23] can be applied either at the server level or at the device (edge computing). If one chooses to upload pictures on the server, one can apply more sophisticated classification models at the endpoint as there are no restrictions on power and hardware, but this makes intense use of costly communication bandwidth and power. If one processes the images on the device, then one can only report results and environmental parameters to the server and reduce significantly the transferred data load because transmission of images dominates the overall energy consumption. In this work, we upload both the picture and the counting results for demonstration and verification, but in operational mode only the counting results and the environmental variables will be transmitted. A lower communication bandwidth requirement allows the use of a long-range, low-power, wide-area network modulation technique (LoRa) that can enlarge the battery life tenfold. We have the vision to establish remote automated monitoring of all insects of economic and hygienic importance at large spatial scales using different modalities according to the application (i.e., optical counters [2,5], camera-based devices [10], spectral [24] and multi-spectral sensors [25]). To achieve this, one needs to prioritize its goals and this prioritization inflicts constraints on the design and accuracy of the system. Our priority is set to present a practical and affordable solution so that it is adopted by the community. Low cost is the first and ultimate policy to meet widespread acceptability. The second priority is power sufficiency and robustness. The devices are spread at large scales and located in cryptic places, usually partially protected against weather conditions. The manual visits to the traps must be therefore sparse and must exceed by a large extent the effective time of replacing a pheromone or a food bait. The third priority is the accuracy of the data reporting procedure and the automatic counting.

This work differs from [10] in that it uses an embedded DL algorithm that counts insects and makes the whole setup more practical by removing the laser beam and introducing DL-based insect counting to identify the entrance of an insect. The novelties of this work with respect to the reported literature are the following: (a) in order to meet the low-cost and low-power requirement we use a microcontroller implementation (ESP32) and we compare it to other low-cost boards, namely RPi4 and Google Coral; the use of a microcontroller with a small amount of memory introduces technical challenges in the design (quantization of deep learning weights, search for optimal structure of the embedded graph) and is programmed in TensorFlow-light; and (b) we include a generic insect counter based on a camera; the counter does not identify the species of insects but only reports their number (i.e., a regression task), and is used as a triggering process to upload a new image and to alert the monitoring procedure in case the inferred number of captured insects surpasses a threshold. The literature on counting insects based on the image is sparse as the main research trend is on localizing and identifying insects.

We believe that if we bring global access to more versatile and more affordable monitoring tools for insects, we will encourage local stakeholders and citizens to engage in the effort of mapping urban insect fauna in their corresponding regions. To this end, we provide at an open source, https://github.com/Gsarant/Edge-computing (accessed date 19 January 2022), the software and models of all electronic components and all necessary details so that they can be freely copied, modified at will, and hopefully massively deployed. Our approach follows the line of thought of [26], where the AudioMoth has brought down tenfold the cost of audio recorders for biodiversity assessment of vocalizing animals and allowed the widespread use of affordable audio monitoring tools, to improve coverage for conservation researchers and managers.

## 2. Materials and Methods

In this section we start with the basic principles of edge devices with wireless communication functionality and a camera. There are many hardware choices we can take to face the task but in order to meet the low-cost, high power-sufficiency we need to go down to the level of microcontrollers such as the ESP32. The use of a microcontroller with a small amount of memory and the need for power sufficiency restrains us from importing sophisticated but large libraries of object detection models with large weights that require substantial computational resources [27,28,29]. In Appendix A we compare ESP32 with other more advanced hardware platforms running the same software and list the technical capabilities and their corresponding costs.

### 2.1. The Edge Devices

**Figure 1:**
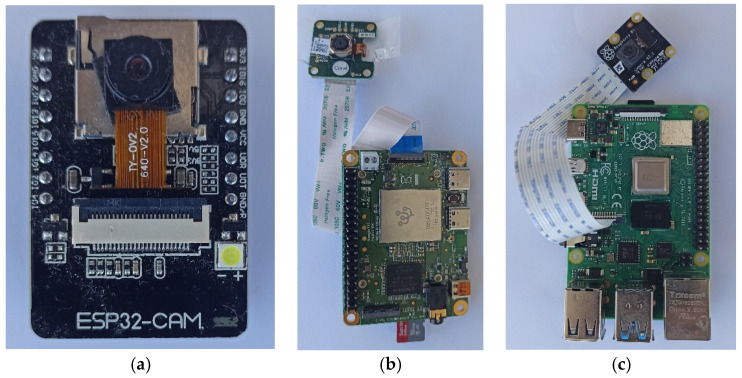
(**a**) The ESP32-CAM-based device, (**b**) the Coral-based device, and (**c**) the Raspberry Pi4 device.

In Figure 1, we present the boards we have tested and compared on the same tasks. Each edge device is equipped with a camera and WiFi communication. All devices run Tensor Flow light. In each memory we embed the same DL model that we have trained off-line after quantization. The size after quantization is 1/10 of the original (see Section 3). Camera quality is a significant factor for camera-based traps. However, in our case, the task is to count the insects and upload a reference image. This is a lighter task than performing species identification or object localization that rely heavily on the quality of the image and allows us to pick more cost-effective solution to suppress the cost. In Appendix A we report the technical details and indicative costs of the cameras we tried.

### 2.2. The Images

**Figure 2:**
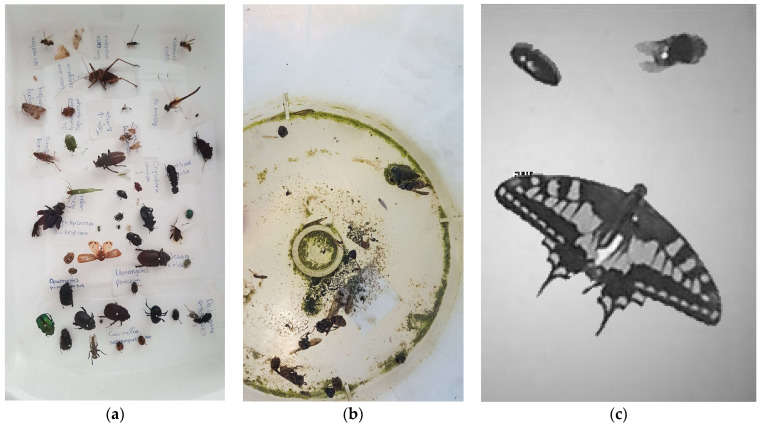
(**a**) A subsample of insects used to make the reference database. (**b**) A typical photo from a funnel trap in the field. (**c**) A photo from the internal space of the suggested device (ESP32-CAM).

In Figure 2a, we present a small sample of the insects used to compose the training set. We do not claim that all these species can be found in urban environments or in this specific trap setup. In fact, we want to build a generic insect counter that is indifferent to the insect species. We need to avoid targeting a specific species if we want to make a device that would count insects in different parts of the globe. Therefore, what we are interested in is to have the maximum diversity of body forms and wing shapes at random poses. In sticky traps like the one used in this work, we do not face the same extent of insect overlap as in traps in agricultural tasks where insects are typically queued in funnels and fall possibly one on top of the other (see Figure 2b). Therefore, our policy for creating the database is to have a variety of insects with very different shapes and forms and to compose many images containing a random number of insects.

The synthesis of pictures with a varying number of insects is also allowing us to have the ground truth of the insects that are depicted in a picture and, therefore, to bypass the very difficult task of manually tagging thousands of insect photos. Figure 2c is an example of a picture taken from the trap in operational conditions.

## 3. Results

### 3.1. Building the Reference Database

The main difficulty of DL applications is not in selecting the model architecture with the right complexity and versatility but gathering the quality and quantity of data needed to train the models. This applies in the case of insects in particular, as they are cryptic creatures and the largest and most diverse group of animals on Earth [30]. The fact that insect biodiversity varies considerably around the globe makes the construction of a generic insect counter harder. Open-source image databases are rare [31] and may refer to a specific targeted insect. Images of insects found on the internet or in online biodiversity databases are not suitable for training devices operating in the field, as they are of high-quality and close-focus, which does not match the pictures taken from the internal space of operational traps in the field (the so-called ‘training-test mismatch’). As shown in Figure 2b, images from traps contain insects at various orientations and degradation level in the presence of debris and varying illumination levels and shadows. In this work we face this challenge by evading the direct collection and tagging of specific species. Our data come from insect collection of students in a department of entomology. One of their graduation duties is to capture, dry, and classify a number of insects found in the field. We extracted 100 different insects from various collections (one insect per species), and we placed them inside the trap randomly and photographed them. We extracted the image from its background and programmed an algorithm that combines them at random numbers. The combined image is superimposed to a background image picked randomly and since the combination number is controlled by the algorithm, the true label corresponding to the true insect counts is known for each composed image and is stored in its filename. The combination is done in a way that avoids significant overlap among pictures. In operational conditions we use sticky traps and, therefore, the probability of significant insect overlap is reduced.

We first place each of the 100 insects inside the trap and we take a photograph of each one alone. 70 of them are retained for the training set and 30 for the test set.

All images for the validation experiments have been created by following an automatic procedure:

1. We take a random image from the folder of backgrounds (null_image.jpg). This folder contains images of backgrounds that differ slightly.
2. We select at random a number between 0–6 and images from the ‘insect for the training set’ folder that matches this random number.
3. Each image is rotated randomly between 0–360 degrees and placed in the background without overlap thus forming a single image. We store the composed image and the reference label (ground truth) of the total number of insects as well.

We repeat steps 1–3 until we create 14,000 images for the training set and 1400 for the test set. For the test set, in step 2 we select randomly from the ‘insects for the test set’ folder as we need to secure that no insect used in the training set is also used in the test set.

**Figure 3:**
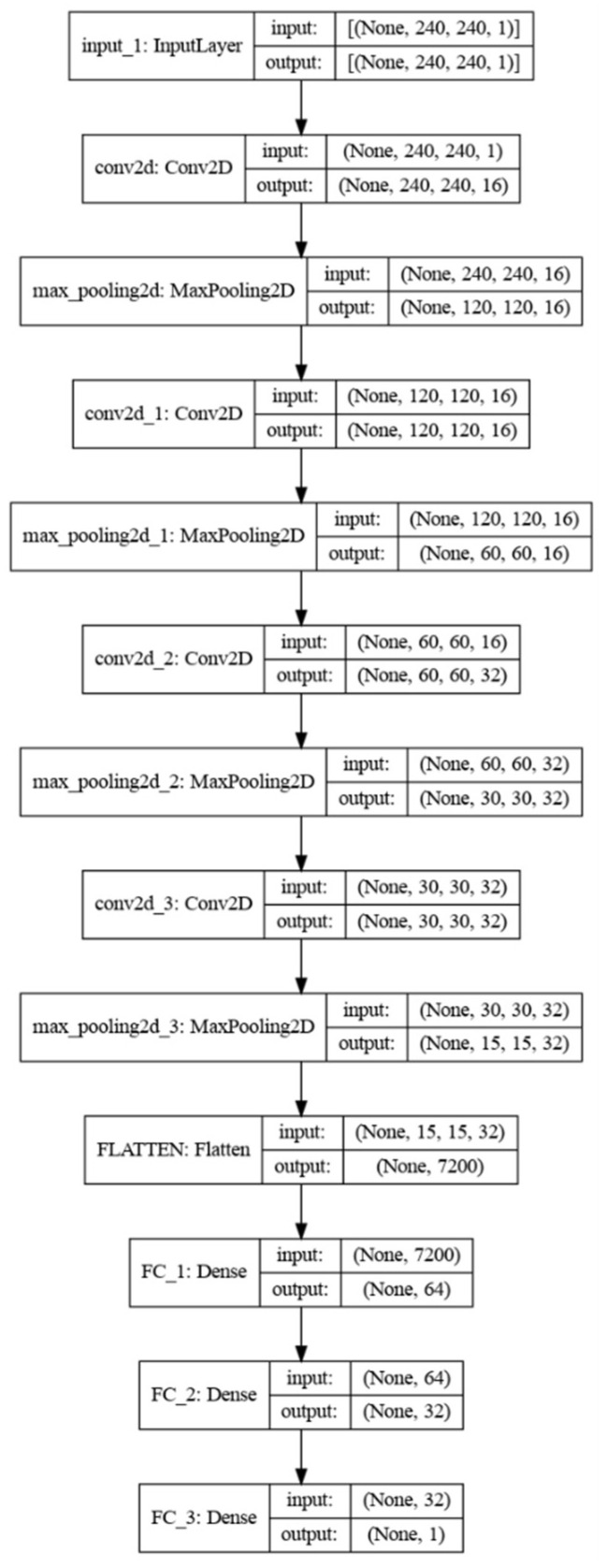
A customized DL model for counting insects that fits into a microprocessor.

We used an 8 CPU, 30 GB RAM, 1 RTX4000 GPU server and the training time was approximately 1 h for training the model in Figure 3 using the database in Table 1. The training procedure evolved smoothly and ran for 240 epochs. It was regulated by the validation set that stopped training at a 0.956 accuracy. The batch size was 32 images. The image size was 240 × 240 pixels grayscale. We picked a mean square error (MSE) training loss as this is a regression task. We tried mean absolute error as a loss function as well, but we did not observe a noticeable difference worth mentioning. The optimizer was Adam with learning rate = 0.001, and weights relaxation (beta_1 = 0.9, beta_2 = 0.999) (see [23]). During training we followed a standard augmentation policy that consists of random flips (horizontal and vertical) with probability 0.5, random rotation with probability 0.5 and random zoom with probability 0.2. The final model is depicted in Figure 3.

### 3.2. Verification Experiments

The absolute constraints imposed by the memory of the systems forced us to create our own model, tailored to the memory size of the smallest device (ESP32), instead of importing a more sophisticated model (Fast R-CNN [27], Inception v3, Yolov4-tiny, VGG-19 etc.). Even models such as SqueezeNet and MobileNet that have been developed for mobile devices [23] and would fit in RPi4 and Coral are too large for the basic ESP32 microcontroller-based system. However, it is possible to optimize a custom neural network architecture to fit within the constraints of a microcontroller [27] without sacrificing accuracy. Once the model in Figure 3 has been trained offline using TensorFlow, the following step is to process it TensorFlow-light and then to quantize the weights at 8bit and form a graph that fits in the memory of the devices. All three hardware platforms report the same accuracy when running the same model. Therefore, we report a single table on accuracy (see Table 2). Note in Table 2 that the weights of the online trained model are 5.9 MB and end up ~0.5 MB after quantization (see also details of the models in Appendix B).

To evaluate the accuracy of the proposed system, we compare the inferred counts of the DL model with the numbers during the composition of the dataset. The inaccuracy of the system is based on the error between the ground truth and system’s prediction. Equation (1), which represents the accuracy of the system, is shown as follows:

α = 1 − |Mc − Ac|/Mc(1)

where α is the counting accuracy of the system, Mc is the true number of insects in an image, and Ac is the number of the automatically counted captures.

As it is clearly presented, our e-trap achieves 95% accuracy on automatic counts compared to the ground truth (see Table 2 and Table 3 for per class accuracy).

In Table 4 we examine another aspect of the hardware platforms, that of the processing time. The ESP32 has, by far, the worst performance compared to RPi4 and Coral devices. The Coral with the TPU accelerator is extremely fast compared to any other hardware platform we examined. However, speed of execution is a quality we are more than willing to sacrifice in the context of this specific task to lower the cost. The e-trap takes only one photo per day, which is enough for insect monitoring applications. Insect monitoring does not need the high frequency rates of video processing and real-time performance. The interested reader needs to see the indicative cost of its hardware platform in Appendix A with ESP32 being the most affordable one at USD 8 (as per 30/12/21, see Table A1) and also power consumption as depicted in Table 5.

Last, we examine the important parameter of power consumption. E-traps offer the benefit of reducing the costs of manual visits to the traps; therefore, they need to be power sufficient for as long as possible. In Table 5, we gather the consumptions of all hardware platforms. Given that a device carries two batteries, 2 × 3350 = 6700 mAH at 3.7 V (3.5–4.2), the ESP32 is expected to last 50 days when uploading one image per day, as well as the classification results.

Note that power sufficiency is measured by using a maximum consumption scenario as the device uploads an image only if there is a difference in the insect counts from day to day. In urban traps, this does not happen often. Similarly, if classification is performed locally on the edge device and only the classification output is transmitted (no photo uploaded), this achieves a lifetime of 52 days.

### 3.3. Operational Conditions

**Figure 4:**
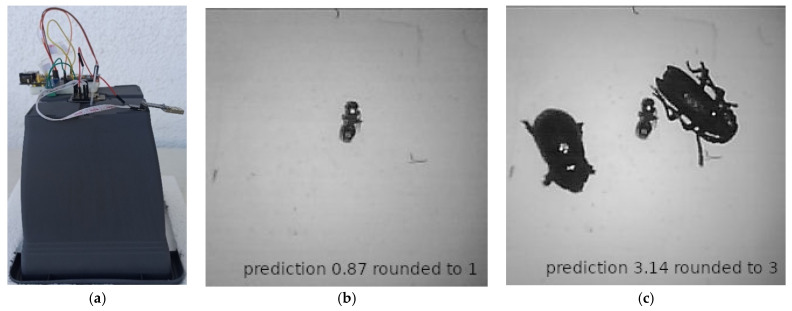
(**a**) The camera-based trap in operational conditions. (**b**) The case of one insect at day T. (**c**) At day T + 1 there are three insects. The difference in inferred counts from day T to T + 1, triggered an alert. Counting does not require a high analysis picture as in an identification task. Note the low quality of the pictures in (**b**,**c**).

During operation (see Figure 4a), the device takes a photo once a day using a flash. If the insect counts predicted differ from the previous count, then the latest photo and the counting results are uploaded to the server through a WiFi connection (see Figure 4b,c). The last picture is stored in the SD card (only for validation, as it is not necessary). The list of tasks can be found below.

All devices carry out the following chain of tasks:

(a) They wake up by following a pre-stored schedule and load the DL model weights;
(b) They take a picture once per day at night with flash;
(c) They infer the number of insects in the picture;
(d) If the insect count in the current picture is different from the previous count, the image is uploaded to a server through WiFi by making an http, post request;
(e) They store the last picture in the SD (non-mandatory);
(f) They go into a deep sleep mode and follow steps (a)–(e).

## 4. Concluding Remarks and Further Steps

Smart cities gradually adopt more sophisticated means to control urban pests that have economic and human health implications [32,33]. We have presented an e-trap that provides consistent estimates not only of insects’ presence (detection) but of relative abundance (monitoring). It is also useful for evaluating insecticide treatment efficacy (post-treatment analysis) and control (population reduction). In this work, we decided to count by regression. This entails that the DL model learns a direct mapping from a picture to countable insects and skips localization, semantic segmentation, and species recognition. We took this approach because the latter tasks are typically carried out by employing larger and more sophisticated models and, in our application, we struggle with memory and power limitations and also because it has been reported that counting by regression is more robust to insect overlapping [34]. The single most important outcome of this paper is that a microcontroller worth USD8 (as per 30 December 2021, see Table A1) can adequately carry out the task of taking and image from inside a trap, apply a DL-based, insect counting model using TensorFlow-light micro and upload the results through its WiFi modem. We traded cost- and power-sufficiency that are of paramount importance with execution speed that is not important in the context of this specific application. Further steps include its mass deployment in a city and the analysis of the feedback from citizen science.

## Figures and Tables

**Table 1:** The reference database. Numbers 0–6 denote how many insects are in an image.

# Images	Training (70%)	Validation (30%)	0	1	2	3	4	5	6
**Train:** 14,000	9800	4200	2000	2000	2000	2000	2000	2000	2000
			**0**	**1**	**2**	**3**	**4**	**5**	**6**
**Test:** 1400			200	200	200	200	200	200	200

**Table 2:** Accuracy and model size in MB for various backends.

Software Back-End	Model Name	Acc. (α = 1 − |Μc − Ac|/Mc)	Model Size (MB)
TensorFlow	model_count_final.h5	0.951	5.9
TensorFlow Lite	model_count_final.tflite	0.951	2
TensorFlow Lite Quantization	model_count_final_quant.tflite	0.950	0.5
TensorFlow Lite Quantization TPU (Coral)	model_count_final_quant_edgetpu.tflite	0.950	0.55

**Table 3:** Accuracy (α = 1 − |Μc − Ac|/Mc) as measured per class. Accuracy drops from 99% for the background class to 95% for the 6 insects class. The mean acc. is 0.950.

Number of Insects Per Image	Accuracy (α = 1 − |Μc − Ac|/Mc)
**0**	0.991
**1**	0.942
**2**	0.931
**3**	0.945
**4**	0.945
**5**	0.953
**6**	0.951
**Mean Accuracy**	0.950

**Table 4:** Processing time for all edge devices on the same quantized model: (a) load models and initialize the inference procedure, (b) process an image and derive a count of the insects inside.

Edge Device	Model	Load Model and Initialize	Inference Time
ESP32-CAM	TensorFlow Lite Quant. Micro	51 s	
Raspberry Pi4	TensorFlow Lite Quant.	70.550 μs	88.868 μs
Coral mini Dev	TensorFlow Lite Quant.	6.726 μs	132.546 μs
Coral mini Dev	TensorFlow Lite Quant. TPU (Coral)	385.854 μs	31.531 μs

**Table 5:** Power consumption for key tasks. First column: Standby and deep sleep Average current. This is the key consumption number that allows the ESP32 to be the suggested solution. Second column: Avg. current: the consumption to take a picture with flash and the time needed to carry out the task. Third column: Avg. current for inference, consumption, and time to run the model for 1 picture. Fourth column: current required to save a picture to the SD and upload it through the WiFi. Fifth column: Avg. current to carry out other functions such as: system initialization, setup WiFi, load model and initialize camera. Last column: Total consumption and time to carry out all tasks from waking up, performing all tasks till going back to standby mode.

Edge Device	Stand By or Deep Sleep Avg. Current (mA)	Avg. Current		Avg. Current Inference		Avg. Current Store in SD Wifi Upload		Avg. Current Other Functions		mA in 63 s
	mA	Sec	mA	Sec	mA	Sec	mA	Sec	mA	mA
ESP32-CAM TensorFlow Lite Micro	6	2	180	51	85	3.5	150	6.5	70	5595
Raspberry pi 4 B TensorFlow Lite	410	0.914	470	0.174	560	0.918	490	60.994	410	25984.38
Coral mini Dev TensorFlow Lite	240	0.062	400	0.135	460	0.08	450	62.723	240	15176.42
Coral mini Dev TensorFlow Lite TPU (Coral)	240	0.062	400	0.036	460	0.076	450	62.826	240	15153.8

## Data Availability

A link to the database used in this work can be found at: https://github.com/Gsarant/Edge-computing, accessed on 19 January 2022.

## Appendix A

**Table A1:** Technical specifications of the hardware platforms compared in this work. The cost is indicative (30/12/21).

	ESP32	Raspberry Pi 4 Model B	Coral Dev Board Mini Datasheet
CPU	Xtensa® dual-core 32-bit LX6 microprocessor(s), up to 600 MIPS 160 MHz	Broadcom BCM2711, quad-core Cortex-A72 (ARM v8) 64-bit SoC @ 1.5GHz	MediaTek 8167s SoC Quad-core ARM Cortex-A35 1.5 GHz
GPU			Imagination PowerVR GE8300
TPU			Google Edge TPU ML accelerator
RAM	520KB SRAM +4M PSRAM	4 Gigabyte LPDDR4 RAM	2 GB LPDDR3
Flash	4 MB Flash		8 GB eMMC,
WiFi	802.11 b/g/n/	802.11 b/g/n/ac Wireless LAN	WiFi 5
Ethernet		Gigabit Ethernet port (supports PoE with add-on PoE HAT)	
Bluetooth	Bluetooth 4.2 BR/EDR BLE	Bluetooth 5.0 with BLE	Bluetooth 5.0
SD Card	TFCard	SD Card	Meets SD/SDIO 3.0 standard
Camera	SCCB	1× Raspberry Pi 2-lane MIPI CSI Camera and 1x Raspberry Pi 2-lane MIPI DSI Display connector	MIPI-CSI2 camera input
Operating System	Free RTOS	Raspbian	Mendel Linux
	Turn off flash lamp 180 mA@5V	Typical 800 mA	Accelerator Module
	TurnOn flash lamp 310 mA@5V	Stress 1200 mA	TPU used 425 mA 212 mA@3,3 V
	Deep-sleep 6 mA@5V	Idling 600 mA	Typical idle 114–121 mA@3,3 V
		Halt current 23 mA	PMIC digital I/O power supply current (AON) 10 mA
Sources	Copyright© 2022 Shenzhen Ai-Thinker Technology Co., Ltd. All Rights Reserved	Raspberry Pi 4 Model B Datasheet Copyright Raspberry Pi (Trading) Ltd. 2019	Dev Board Mini datasheet
	US$7.99	$55.00	$110.95
	OV2640	Raspberry Pi Camera Module	Coral Camera
Chip	OV2640 CAMERA CHIP	Sony IMX 219 PQ CMOS image sensor in a fixed-focus module.	5-megapixel OmniVision sensor
Resolution	2-megapixel	8-megapixel	5-megapixel
Max Resolution	1600 × 1200	3280 × 2464	2582 × 1933
Video	UXGA(1600 × 1200) 15fps	1080p(1920 × 1080) 30fps	
	SVGA(800 × 600) 30fps	720p(1280 × 720) 60fps	
	CIF (352 × 288) 60fps	(640 × 480) 60/90 fps	
Image area	3.59 × 2.684 mm	3.68 × 2.76 mm (4.6 mm diag.)	2.5 mm
Pixel size	2.2 μm × 2.2 μm	1.12 µm × 1.12 µm	1.4 × 1.4 μm pixel size
Picture formats	YUV(422/420) YCbCr422, RGB565/555, 8-bit Compressed data, 8~10 bit Raw RGB data	JPEG, JPEG + DNG (raw), BMP, PNG, YUV420, RGB888	
Len Size	1/4″	1/4″	1/4″
Sensitivity	600 mV /Lux-sec	680 mV/lux-sec	
Cam Cost	$7.99 camera and board	$25.00	$24.95

## Appendix B

Code and images and links to a database can be found at: https://github.com/Gsarant/Edge-computing accessed on 19 January 2022 at the corresponding folders. Regarding the trained weights we comment as follows:

1. model_count_insects_final.h5. The model that was trained offline.
2. model_count_insects_final.tflite a smaller version of model_count_bugs_final.h5 to be executed by Tensorflow Lite.
3. model_count_insects_final_quant.tflite a smaller version of model_count_bugs_final.h5 to be executed by Tensorflow Lite but is now quantized (8 bit).
4. model_count_insects_final_quant.cc a matrix with structure and quantized weights in C to work with Tensorflow Lite micro to esp32-cam.
5. model_count_insects_final_quant_edgetpu.tflite This is the Coral version of model_count_insects_final_quant.tflite that has been processed to run in Coral’s TPU.
